# The genotoxicity of an aqueous extract of Gyejibokryeong-hwan

**DOI:** 10.1186/s12906-017-2054-z

**Published:** 2018-01-22

**Authors:** Mee-Young Lee, Chang-Seob Seo, Hyekyung Ha, Eunsook Park, Ji-Young Kim, Hyeun-Kyoo Shin

**Affiliations:** 1 K-herb Research Center, Korea Institute of Oriental Medicine, 1672 Yuseongdae-ro, Yuseong-gu, Daejeon, 305-811 Republic of Korea; 2 Division of Nonclinical Studies, Korea Institute of Toxicology, P.O. Box 123, 19 Sinseongro, Yuseong-gu, Daejeon, 305-343 Republic of Korea

**Keywords:** Gyejibokryeong-hwan, Ames test, Chromosome aberration assay, Micronucleus

## Abstract

**Background:**

Gyejibokryeong-hwan (*Guizhi Fuling Wan* in China), a mixture of five herbal plants, is a well-known treatment for renal diseases including those associated with climacteric syndrome. However, the genotoxicity of Gyejibokryeong-hwan has not yet been well established.

**Methods:**

The present study investigated that the genotoxicity of an aqueous extract of Gyejibokryeong-hwan (GJBRHE): an in vitro chromosomal aberration test using Chinese hamster lung cells, an in vitro bacterial reverse mutation assay (Ames test) with *Salmonella typhimurium* and *Escherichia coli* strains, and an in vivo micronucleus test using ICR mouse bone marrow.

**Results:**

GJBRHE with or without the S9 mix showed no genotoxicity in the Ames test up to 5000 μg/plate or in the in vivo MN test up to 2000 mg/kg body weight. In contrast, the chromosomal aberration test showed that GJBRHE induced an increase in the number of chromosomal aberrations compared with the control after treatment for 6 h with 4200 μg/mL GJBRHE in the presence of the S9 mix and for 22 h with 800 μg/mL GJBRHE in the absence of the S9 mix.

**Conclusions:**

GJBRHE did not cause detectable genotoxic effects in the bacterial mutation test or the in vivo MN test, however genotoxic effect was detected in the in vitro chromosomal aberration assay. Our results suggest that GJBRHE may be associated with a low risk of carcinogenesis. Thus, further detailed experiments would be needed to clarify the compound responsible for inducing this genotoxicity of GJBRHE and to determine its mechanism.

## Background

Many herbal medicines are widely used to be safety materials than other medicines [1, 2]. The use of herbal products as first choice in primary supplements for improving health is popular [3]. Because people have become more interested in safety and in their well-being [4]. Recently, concerns have been raised the scientific evidence for the safety and efficacy of these herbal medicines [5, 6]. However, the safety information of herbal medicines including oral toxicity, genotoxicity has been not yet enough. Among them, a genotoxicity test includes the hazard identification with regard to DNA damage and is required for the development of new drug [7].

Gyejibokryeong-hwan (Guizhi Fuling Wan in China and Keishi-bukuryo-gan in Japan) is a traditional Korean herbal formula consist of five medicinal herbs: *Cinnamomum cassia*, *Poria cocos*, *Oaeonia lactiflora*, *Paeonia suffruticosa*, and *Prunus persica*. Gyejibokryeong-hwan has been used to treat climacteric symptoms caused by blood stasis and mass uterine disorders [8, 9]. It has been reported that Gyejibokryeong-hwan exhibits biological and pharmacological activities against inflammation [10], cardiovascular diseases [11], diabetes [12], diabetic nephropathy [13], brain ischemia [14], and various cancers [4, 15]. However, genotoxicity has not yet been studied. To ensure safety of the Gyejibokryeong-hwan, the current study performs genotoxicity assessment. The aim of a genotoxic assay is to detect carcinogens and other mutagens [16]. Therefore, the present study was aimed to determine the acute toxicity and genotoxic properties of an aqueous extract of Gyejibokryeong-hwan (GJBRHE) in assays including a bacterial reverse mutation (Ames) test, a chromosome aberration test, and an in vivo micronucleus (MN) test.

## Methods

### Chemicals and reagents

**Fig. 1:**
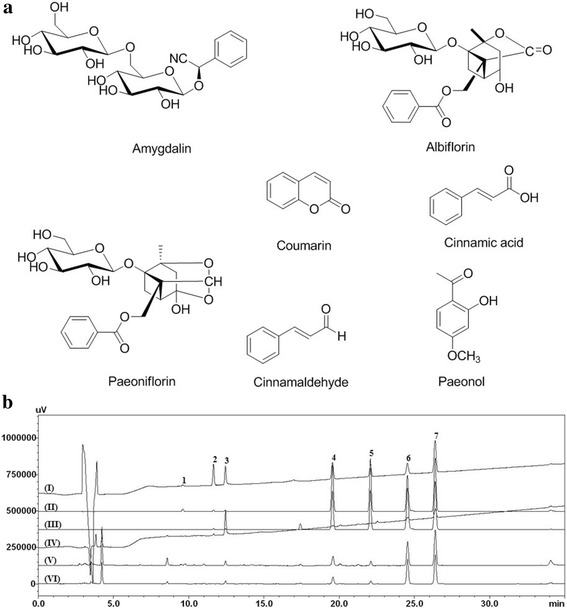
HPLC chromatograms of (**a**) the reference standard mixture and (**b**) GJBRHE: 230 nm (I), 254 nm (II), and 280 nm (III)

Reference standards of amygdalin, coumarin, and cinnamic acid were purchased from Sigma-Aldrich (St. Louis, MO, USA). Albiflorin, cinnamaldehyde, paeoniflorin, and paeonol were obtained from Wako (Osaka, Japan). The purity of the seven reference standards was ≥98.0%. The chemical structures of the seven marker components are shown in Fig. 1a. High-performance liquid chromatography (HPLC)-grade reagents methanol, acetonitrile, and water to obtain the aqueous extract of GJBRHE were obtained from J. T. Baker (Phillipsburg, NJ, USA). Acetic acid was purchased from Merck (Darmstadt, Germany).

### Plant materials

The component herbs of Gyejibokryeong-hwan (*Poria cocos*, *Paeonia suffruticosa*, *Cinnamomum cassia*, *Paeonia lactiflora*, and *Prunus persica*) were purchased from Kwangmyungdag Medicinal Herbs (Ulsan, Korea) in November 2013 and identified by Prof. Je Hyun Lee (Department of Herbology, College of Oriental Medicine, Dongguk University, Gyeongju, Korea). Voucher specimens (2013–KE31–1 to KE31–5) have been deposited at the K-herb Research Center, Korea Institute of Oriental Medicine (KIOM).

### Preparation of Gyejibokryeong-hwan decoction

To obtain the Gyejibokryeong-hwan aqueous extract (GJBRHE), the five herbal medicines comprising Gyejibokryeong-hwan were mixed as shown in Table 1 (total weight = 125.0 kg, equivalent to approximately 666.7 single doses), and extracted in a 10-fold mass of water (1250 L) at 100 °C for 2 h. The aqueous extract was filtered by pressing through a filter (10 μm pore size), and then the solution was evaporated and freeze-dried to give a powder. The amount of Gyejibokryeong-hwan decoction powder was 15.7 kg (12.56%). For HPLC analysis, 400 mg of lyophilized Gyejibokryeong-hwan decoction powder was dissolved in 20 mL of distilled water and then extracted by sonication for 10 min on room temperature. The solution was filtered through a 0.2 μm pore-size syringe filter (Woongki Science, Seoul, Korea) before HPLC injection.

**Table 1:** The combination of crude components of Gyejibokryeong-hwan aqueous extract (GJBRHE)

Herbal name	Scientific name	Family	Used parts	Original region	Amount (g)
Cinnamomi Ramulus	*Cinnamomum cassia* Presl	Lauraceae	Ramulus	Vietnam	3.75
Hoelen	Poria cocos Wolf	Polyporaceae	Sclerotium	Pyeongchang, Korea	3.75
Moutan Cortex Radicis	*Paeonia suffruticosa* Andrews	Paeoniaceae	Bark	Jecheon, Korea	3.75
Paeoniae Radix	*Paeonia lactiflora* Pallas	Paeoniaceae	Root	Uiseong, Korea	3.75
Persicae Semen	*Prunus persica* Batsch	Rosaceae	Seed	South Africa	3.75
Total amount					18.75

### HPLC analysis of GJBRHE

The chromatographic analysis for quantitative determination of the seven marker components in Gyejibokryeong-hwan was performed using a Shimadzu Prominence LC-20A system (Shimadzu Co., Kyoto, Japan) consisting of a solvent delivery unit, an on-line degasser, a column oven, a sample autoinjector, and a photodiode array (PDA) detector. The data were acquired and processed using LCsolution software (Version 1.24). The column used for separation of the seven constituents was a Phenomenex Gemini C_18_ column (250 × 4.6 mm, 5 μm, Torrance, CA, USA) maintained at 40 °C. The mobile phases consisted of water (A) and acetonitrile (B), both containing 1.0% (*v*/v) acetic acid. The gradient elution of the mobile phase was as follows: 10–60% B for 0–30 min, 60–100% B for 30–40 min, 100% B for 40–45 min, 100–10% B for 45–50 min, and 100% B for 50–60 min. The flow rate and injection volume were 1.0 mL/min and 10 μL, respectively. The detection wavelengths for quantitative analysis of the seven compounds were set according to the maximum absorption wavelengths of each reference compound (amygdalin, albiflorin, paeoniflorin for *P. persica*, coumarin, cinnamic acid, cinnamaldehyde for *C.cassia*, paeonol for *P.suffruticosa*)..

### Acute oral toxicity study

This study was approved by the Institutional Animal Care and Use Committee (IACUC) of Korea Institute of Toxicology (KIT; Daejeon, Korea); it was performed at KIT and was conducted according to the guidelines of the KIT IACUC (Number: G13088), which is accredited by the Association for Assessment and Accreditation of Laboratory Animal Care International (1998) under the Good Laboratory Practice Regulations for Nonclinical Laboratory Studies. Specific-pathogen-free Sprague Dawley rats (5 males and 5 females) were purchased from Orient Bio Co. (Seongnam, Korea). The acute oral toxicity study in rats was conducted as described previously [17]. Our preliminary study (N:P13030) showed that a single oral administration of GHBRHE did not induce any toxic effect at dose levels up to 5000 mg/kg/day (data not shown). Based on this study, a dose of 5000 mg/kg/day was selected as the experimental limit dose. GJBRHE was suspended in distilled water. The vehicle control rats received distilled water only. All animals were observed for 14 days and mortality, body weight changes, clinical signs, and gross findings were recorded. This study was conducted in accordance with the guidelines established by the FDA Good Laboratory Practice (GLP) for Nonclinical Laboratory Studies (21 CFR Part 58) and OECD guidelines [18].

### Ames test

The used experimental methods were based on the report of Maron and Ames [19], with minor modifications. The Ames test was conducted as described previously [17]. Distilled water was selected as the vehicle based on the results of the solubility test and aseptic test. Various concentrations of GJBRHE were incubated with tester strains in the presence or absence of metabolic activation with the S9 mix along with vehicle. Each concentration of GJBRHE was assayed in triplicate. The present study was conducted in accordance with OECD guidelines for the Testing of Chemicals Section 4 Health Effects Test No. 471 about Bacterial Reverse Mutation Test (21 July 1997).

### Chromosome aberration test in Chinese hamster lung (CHL) cells

Chromosome aberration tests were conducted as described previously [17] with minor modifications as described by Ishidate [20] and by Dean and Danford [21]. Chinese hamster lung (CHL) cells were selected as the test system because they are sensitive to mutagens, their low chromosome number facilitates scoring and the same cell line can be used repeatedly. Furthermore, because CHL cells are a widely used test system for in vitro mutagenicity studies, a broad historical database exists. The modal chromosome number of this cell line is 25 with a doubling time of ~15–17 h. The cells are thawed in culture medium and then grown for more than 7 days as a monolayer. Sterility was checked by inverted microscopy to determine any gross mycoplasma contamination and confirmed by polymerase chain reaction amplification. Cells were cultured in reconstituted modified Eagle’s medium (Gibco-Invitrogen, Carlsbad, CA, USA) supplemented with Na_2_HCO_3_ (2.2 g), l-glutamine (292 mg), streptomycin sulfate (100 μg), penicillin G·Na (10^5^ units) and 10% (*v*/v) fetal bovine serum (Gibco-Invitrogen) per liter. The cultures were incubated at 37 °C under humidified 1.5% CO_2_ in air. Based on the results, the dose range for the present study was designed considering the solubility and cytotoxicity of SST. Ethyl methanesulfonate (EMS) was used as a positive control chemical without metabolic activation using the S9 mix, and cyclophosphamide (CPA) was used as a positive control with metabolic activation. The present study was conducted in accordance with OECD guidelines for the Testing of Chemicals Section 4 Health Effects Test No. 473 about In vitro Mammalian Chromosome Aberration Test (21 July 1997).

### In vivo MN test

The MN test was performed as described by Muangphra and Gooneratne [22] with slight modifications. Bone marrow preparations were made according to the method described by Schmid [23]. MN tests using ICR male mice were conducted as described previously [17, 24]. The preliminary study showed that oral administration of GJBRHE did not induce any toxic effect at 2000 mg/kg (the dose limit for treatment up to 14 days according to OECD guidelines). This was selected as the maximum dose. Specific-pathogen-free male ICR strain mice weighing 27.2–30.0 g were obtained from Orient Co., Ltd. (Seongnam, Korea) at 6 weeks of age. According to Schmid [23] method, ICR mice were euthanized by CO_2_ gas after the final administration of GJBRHE and bone marrow preparations were made. Small round or oval bodies, measuring approximately 1/5 to 1/20 the diameter of a polychromatic erythrocyte (PCE), were counted as micronuclei. A total of 2000 PCEs were scored per animal to determine the frequency of MNPCEs and PCE/(PCE + NCE) ratio was calculated by counting 500 cells. Differences in the numbers of MNPCEs between treatment and control groups were analyzed using the Kruskal–Wallis *H* test and Dunn’s rank sum test. The Mann–Whitney nonparametric *U* test was used to compare the PCE/(PCE + NCE) ratios of treatment and control groups. Significance for all tests was accepted at *P* < 0.05.

This study was approved by the Institutional Animal Care and Use Committee (IACUC) of Korea Institute of Toxicology (KIT; Daejeon, Korea); it was performed at KIT and was conducted according to the guidelines of the KIT IACUC (Number: N14005), which is accredited by the Association for Assessment and Accreditation of Laboratory Animal Care International (1998) under the Good Laboratory Practice Regulations for Nonclinical Laboratory Studies.

### Statistical analyses

Acute toxicity study, a one-way analysis of variance was performed at α = 0.05 in cases in which Bartlett’s test indicated no significant deviations from variance homogeneity. When significance was noted, a multiple-comparison test (Dunnett’s test) was used to determine which pairs of groups were significantly different. Statistical analyses were performed using the PATH/TOX system. Chromosome aberration assay, the statistical analyses were based on the methods used in the published reports [25] using Statistical Analysis System (SAS) software. Each metaphase was classified as a normal metaphase or an aberrant metaphase with one or more aberrations, and the frequency of aberrant metaphases was analyzed statistically. The χ^2^ test and Fisher’s exact test [26] were performed to compare the vehicle control and GJBRHE-treated groups. The in vivo MN results were evaluated as described previously [27] using the method of Lovell et al. [28] with minor modifications. Data with heterogeneous variances were analyzed using Kruskal–Wallis analysis of variance followed by multiple comparisons using Dunnett’s test [29]. The significance was accepted when all of the PCE/(PCE + NCE) ratios were > 0.1. The result was judged as positive when there was a significant and dose-related increase or a reproducible increase in the frequency of MNPCEs or aberrant metaphases at one or more dose levels. Differences were regarded as significant at *P* < 0.05.

## Results

### Quantitative analysis of the seven marker compounds in Gyejibokryeong-hwan

The optimized HPLC–PDA method was used for quantitative determination of the seven marker compounds in the Gyejibokryeong-hwan sample. Using optimized chromatography conditions, all analytes separated in under 30 min; a typical HPLC chromatogram is shown in Fig. 1b. The wavelength of the PDA ranged from 190 to 400 nm and the detection wavelengths for quantitative analysis were 262 nm (amygdalin), 230 nm (albiflorin and paeoniflorin), and 280 nm (coumarin, cinnamic acid, cinnamaldehyde, and paeonol). The correlation coefficient (*r*^2^) of the five compounds showed good linearity of ≥ 0.9999. The retention times of the seven components, coumarin, amygdalin, paeoniflorin, albiflorin, cinnamic acid, cinnamaldehyde, and paeonol were 9.60, 14.80, 15.55, 19.56, 22.08, 24.53, and 26.37 min, respectively. The amounts of the seven marker compounds were 0.52–45.07 mg/g (Table 2). Among the compounds, paeoniflorin, the marker compound of *P. lactiflora*, was detected as the main component of GJBRHE at 45.07 mg/g. This established HPLC–PDA method will be useful for improvement of the quality control of Gyejibokryeong-hwan.

**Table 2:** Amounts of the seven marker compounds in the Gyejibokryeong-hwan aqueous extract by HPLC (*n* = 3)

Compound	Mean (mg/g^*^)	SD × 10–1	RSD (%)	Source
Amygdalin	12.51	2.13	1.71	*P. persica*
Albiflorin	2.29	0.21	0.94	*P. lactiflora*
Paeoniflorin	45.07	2.78	0.62	P. lactiflora
Coumarin	2.62	0.02	0.07	*C. cassia*
Cinnamic acid	0.52	0.01	0.26	C. cassia
Cinnamaldehyde	4.22	0.06	0.14	C. cassia
Paeonol	8.77	0.09	0.10	*P. suffruticosa*

^a^A mount of each compound vs freerzed dried GJBRHE g

### Acute oral toxicity

**Fig. 2:**
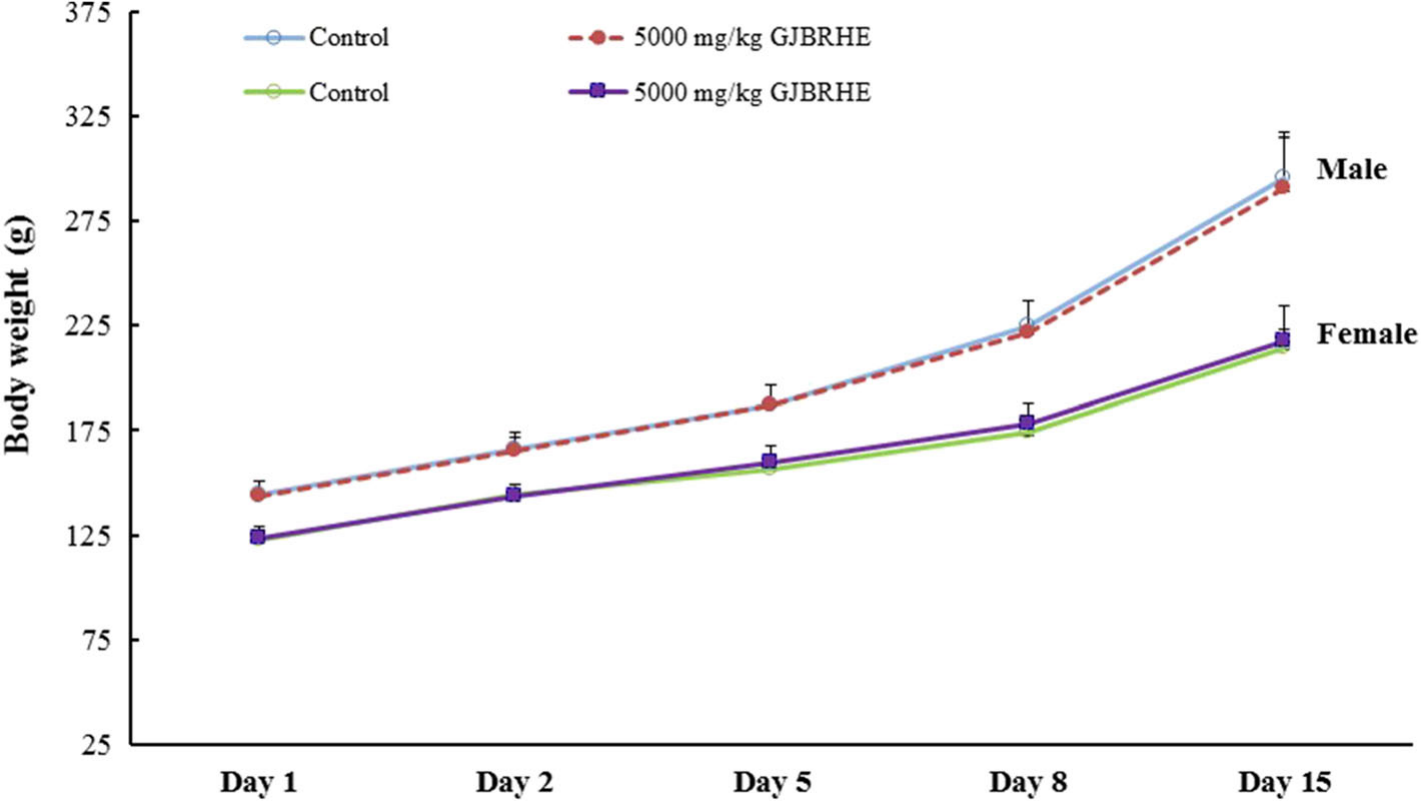
Mean body weight changes after administration of GJBRHE

During the experimental period, no clinical symptoms or no mortality of toxicity were observed in rats of either sex in any of the GJBRHE-treated groups during the 14-day observation period. The changes in body weight are shown in Fig. 2. For both sexes, the changes in body weight did not differ significantly between the treated rats with 5000 mg/kg/day of GJBRHE and the control group. At the time of the scheduled autopsy, there were no abnormal pathological observations for the lung, thymus, heart, liver, stomach, adrenals, and spleen in the male or female rats given 5000 mg/kg/day of GJBRHE.

### Ames test

**Fig. 3:**
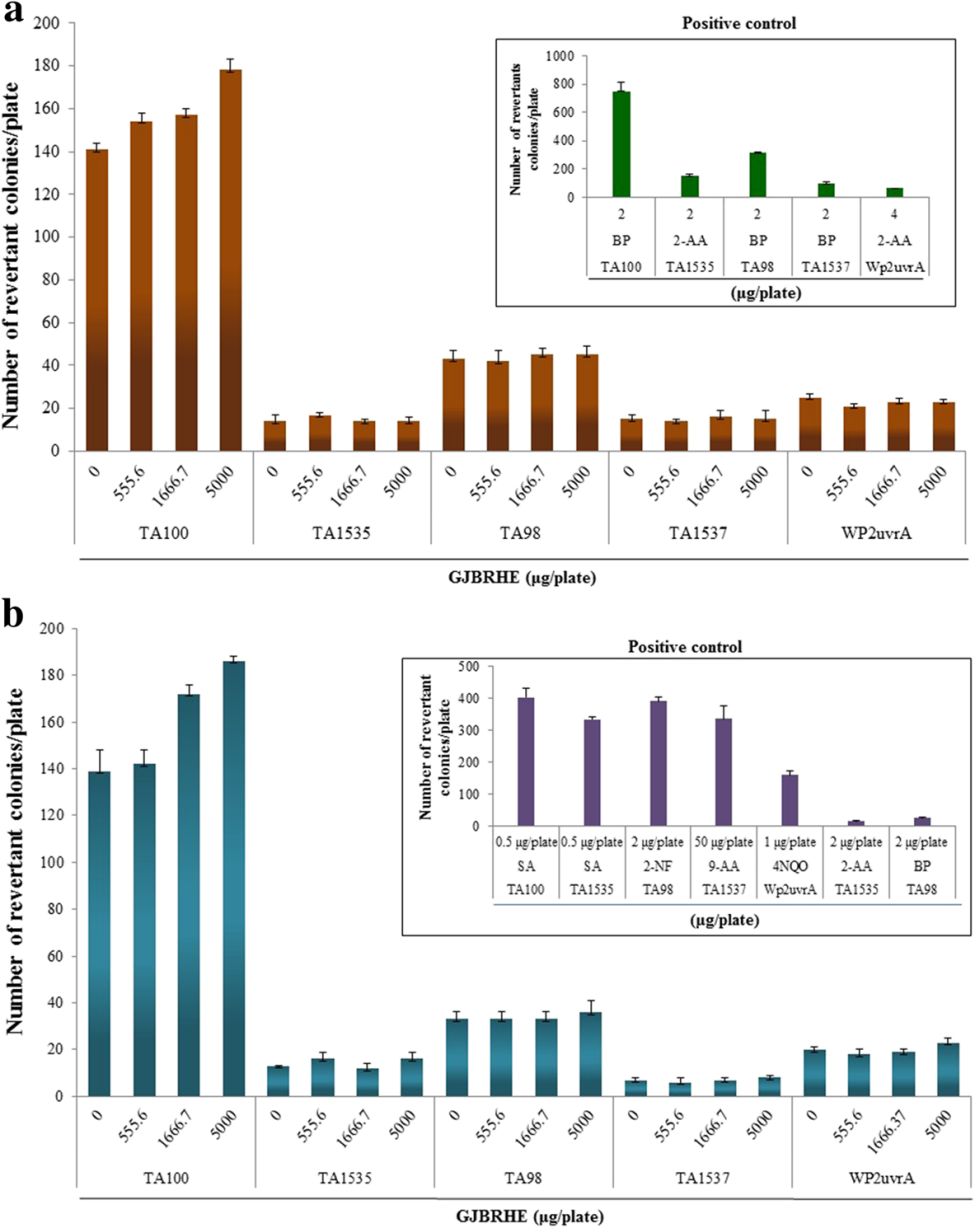
Effects of GJBRHE in the bacterial reverse mutation assay (Ames test) (**a**) in the presence of (+S9 fraction) and (**b**) in the absence of (–S9 fraction) metabolic activation

No positive mutagenic response when compared with concurrent vehicle control groups was observed in any of the *S. typhimurium* TA1535, TA1573, TA100, and TA98 or *E. coli* WP2 *uvr*A strains at any dose level of GJBRHE regardless of the presence (Fig. 3a) or absence (Fig. 3b) of the S9 fraction at up to 5000 μg/plate. There was a significant increase in all positive control groups (Fig. 3a and b).

### Chromosome aberration tests in CHL cells

There were significant increases the frequency of metaphase cells with structural aberrations in cells treated for 6 h after S9 fraction with 4200 μg/mL GJBRHE in the presence of the S9 fraction and in those treated for 22 h S9 fraction with 800 μg/mL GJBRHE without the S9 fraction (Table 3). The positive controls showed significant increases in the frequency of metaphases (Table 3).

**Table 3:** Chromosome aberration assay and relative cell counts of Gyejibokryeong-hwan aqueous extract (GJBRHE)

Nominal conc. of GJBRHE (μg/mL)	S9 mix	Time hours ^a)^	Mean total aberrant metaphases	Mean total aberrations	Mean of PP + ER	Relative cell counts (%)
6 h treatment (+S9)						
0	+	6–18	1.0/0.5^b)^	1.0/0.5	0.0 + 0.0	100
1050 ^#^	+	6–18	0.5/0.5	0.5/0.5	1.0 + 0.0	98
2100 ^#§^	+	6–18	1.5/0.5	1.5/0.5	0.0 + 0.0	80
4200 ^#§^	+	6–18	21.0/21.0^**^	36.0/36.0	1.5/0.0	47
4500 ^#§^	+	6–18		Not counted		43
CPA 6	+	6–18	24.0/24.0^**^	38.0/37.5	0.5 + 0.0	63
6 h treatment (-S9)						
0	–	6–18	0.5/0.0	0.5/0.0	0.0 + 0.0	100
225	–	6–18	Not counted			94
450 ^#§^	–	6–18	1.5/1.0	1.5/1.0	1.5 + 0.0	80
900 ^#§^	–	6–18	1.0/1.0	1.0/1.0	0.0 + 0.0	66
1050 ^#§^	–	6–18	2.0/0.5	2.0/0.5	1.0 + 0.0	50
EMS 800	–	6–18	25.5/25.0^**^	37.0/36.0	0.0 + 0.0	58
22 h treatment (-S9)						
0	–	22–2	1.0/1.0	2.0/2.0	0.5 + 0.0	100
180	–	22–2	1.5/1.0	1.5/1.0	0.0 + 0.0	89
360 ^#§^	–	22–2	2.5/1.5	4.0/2.0	0.5 + 0.0	79
720 ^#§^	–	22–2	Not counted			66
800 ^#§^	–	22–2	7.5/6.5**	9.5/7.5	1.0 + 0.0	48
EMS 600	–	22–2	43.5/43.0**	69.5/68.5	0.0 + 0.0	41

^#^Visible turbidity of test item was observed at the beginning of treatment

^§^Visible turbidity of test item was observed at the end of the treatment

^**^Significantly different from the control at *P* < 0.01

^a)^Treatment time-recovery time

^b)^Gaps included/excluded, means of duplicate cultures; 100 metaphases were examined per culture

### In vivo MN test

No clinical signs and no deaths of toxicity were observed in any of the GJBRHE treatment groups. As shown in Table 4, there was no significant change in body weight compared with the vehicle control between GJBRHE at 500, 1000, or 2000 mg/kg. The cytotoxicity indices, i.e. the PCE/(PCE + NCE) ratios, were 0.59, 0.53, 0.55, and 0.48 for the vehicle control (0), 500, 1000, and 2000 mg/kg/day groups, respectively. The frequencies of micronucleated PCEs among 2000 PCEs were 1.67, 0.67, 0.33, and 0.67 for the vehicle control (0), 500, 1000, and 2000 mg/kg/day groups, respectively (Table 5). There was no significant increase in the frequencies of micronucleated PCEs at any dose level of GJBRHE. The positive control substance induced a significant increase in the ratio, to 54.33 compared with that of the vehicle controls (*P* < 0.01).

**Table 4:** Body weight changes of Micronucleus test in mice following administration of Gyejibokryeong-hwan aqueous extract (GJBRHE)

0	500	1000	2000	70
33.7 ± 0.85	34.2 ± 1.07	33.9 ± 1.54	34.0 ± 1.07	33.2 ± 1.87
34.3 ± 0.66	34.8 ± 0.75	34.0 ± 1.39	34.6 ± 0.87	34.8 ± 1.82
34.4 ± 0.60	35.0 ± 0.81	33.7 ± 0.85	34.8 ± 1.82	33.9 ± 1.69

**Table 5:** Results of micronucleus assay in male mice

0	500	1000	2000	70
1.67 ± 0.58	0.67 ± 1.15	0.33 ± 0.58	0.67 ± 1.15	54.33 ± 6.43
0.59 ± 0.09	0.53 ± 0.08	0.55 ± 0.09	0.48 ± 0.02	0.47 ± 0.05
3	3	3	3	3

*MNPCE* PCE with one or more micronuclei, *PCE* polychromatic erythrocyte, *NCE* normochromatic erythrocyte

Positive control: Cyclophosphamide Monohydrate

## Discussion

GJBRHE comprises five herbs, *Cinnamomum cassia*, *Poria cocos*, *Paeonia suffruticosa*, *Oaeonia lactiflora*, and *Prunus persica*, in 1:1:1:1:1 proportions. The main medicinal constituents of each herb are as follows: amygdalin from *P. persica* [30], coumarin (e.g. cinnamic acid, coumarin, and cinnamaldehyde) from *C. cassia* [31, 32], monoterpenoids (e.g. paeoniflorin and albiflorin) from *Pa. lactiflora* [30] and cyanoglucosides (e.g. amygdalin) from *Pr. Persica* [30]. We analyzed seven of these compounds using HPLC–PDA: albiflorin (2.29 mg/g), amygdalin (12.51 mg/g), coumarin (2.62 mg/g), cinnamic acid (0.52 mg/g), cinnamaldehyde (4.22 mg/g), paeonol (8.77 mg/g), and paeoniflorin (45.07 mg/g). The established HPLC–PDA method was applied for simultaneous analysis of the seven compounds in GJBRHE and detected paeoniflorin (45.07 mg/g), a marker of *G. jasminoides*, as the major component of GJBRHE.

Previous several studies have reported the pharmacological efficacy of Gyejibokryeong-hwan [10–15], but have not provided information about its safety. To evaluate some potential genotoxicities of GJBRHE, we performed an in vitro chromosomal aberration test, an in vitro Ames test, and an in vivo MN test. The results of the present study demonstrated that GJBRHE, a traditional Koran herbal medicine, was not genotoxic using an in vitro Ames test or an in vivo MN test, but genotoxicity was detected in an in vitro chromosomal aberration test.

The in vitro chromosome aberration test is used to identify structural chromosomal aberrations [20]. In present study, there were significant increases the number of metaphases with structural aberrations after treatment for 6 h with GJBRHE at 4200 μg/mL in the presence of the S9 fraction and after treatment for 22 h with GJBRHE at 800 μg/mL without the S9 fraction. However, to confirm these results, more reliable tests are needed.

The Ames test was developed by Ames and coworkers in the early 1970s to assess the mutagenic potential and to estimate carcinogenic potential of environmental mixtures [33]. The Ames test uses strains of *S. typhimurium* TA100, TA98, TA1535, and TA1537 and the tryptophan auxotroph strain *E. coli* WP2 *uvr*A to detect point mutations involving substitution, addition, or deletion of one or more DNA base pairs [19]. The Ames test is commonly employed as an initial screen for genotoxicity, particularly point mutation induction activity. Mutation of genes results in a deficient DNA repair system and greatly enhances the sensitivity of these strains to certain mutagens [34]. There were no increases in the number of revertant colonies of *S. typhimurium* (TA100, TA 98, TA1535, and TA1537) and *E. coli* (WP2 *uvr*A) at any concentration (5000, 1666.7, or 555.6 μg/plate) of GJBRHE in the presence or absence of a metabolic system (S9 fraction). Our results indicate that under the conditions of this study, GJBRHE did not show mutagenicity in the Ames test. Therefore, GJBRHE did not appear to mutate any genes in vitro.

The MN test is useful for the detection of chemically induced chromosome damage [35]. In the present study, there was no significant or dose-related increase in the number of micronucleated PCEs per 2000 PCEs at any GJBRHE treatment dose level. In addition, no abnormal signs in the general appearance and body weight of mouse were observed in any of the GJBRHE treated groups. The PCE/(PCE + NCE) ratio, an indicator of cytotoxicity, was not significantly decreased compared with the vehicle control. An elevated frequency of micronucleated PCEs indicates chromosomal damage [36–38]. MN assay has revealed the accumulated genotoxic damage during the lifetime of the cells [39]. An increased MN frequency is related to cancer risk [40, 41].

## Conclusions

GJBRHE did not cause detectable genotoxic effects in the bacterial mutation test or the in vivo MN test, however genotoxic effect was detected in the in vitro chromosomal aberration assay. Our results suggest that GJBRHE may be associated with a low risk of carcinogenesis. Thus, further detailed experiments would be needed to clarify the compound responsible for inducing this genotoxicity of GJBRHE and to determine its mechanism.
